# Catalyzing Precision Medicine: Artificial Intelligence Advancements in Prostate Cancer Diagnosis and Management

**DOI:** 10.3390/cancers16101809

**Published:** 2024-05-09

**Authors:** Ali Talyshinskii, B. M. Zeeshan Hameed, Prajwal P. Ravinder, Nithesh Naik, Princy Randhawa, Milap Shah, Bhavan Prasad Rai, Theodoros Tokas, Bhaskar K. Somani

**Affiliations:** 1 Department of Urology and Andrology, Astana Medical University, Astana 010000, Kazakhstan; ali-ma@mail.ru; 2 Department of Urology, KMC Manipal Hospitals, Mangalore 575001, India; zeeshanhameedbm@gmail.com; 3 Department of Urology, Kasturba Medical College, Mangaluru, Manipal Academy of Higher Education, Manipal 576104, India; prajwal.ravinder@manipal.edu; 4 Department of Mechanical and Industrial Engineering, Manipal Institute of Technology, Manipal Academy of Higher Education, Manipal 576104, India; bhaskarsomani@yahoo.com; 5 Department of Mechatronics, Manipal University Jaipur, Jaipur 303007, India; princy.randhawa@jaipur.manipal.edu; 6 Department of Urology, Aarogyam Hospital, Ahmedabad 380014, India; drmilapshah@gmail.com; 7 Department of Urology, Freeman Hospital, Newcastle upon Tyne NE7 7DN, UK; urobhavan@gmail.com; 8 Department of Urology, Medical School, University General Hospital of Heraklion, University of Crete, 14122 Heraklion, Greece; ttokas@yahoo.com; 9 Department of Urology, University Hospital Southampton NHS Trust, Southampton SO16 6YD, UK

**Keywords:** prostate cancer, prostate reconstruction, PCa detection, PCa reconstruction, artificial intelligence, deep learning, MRI, PET/CT, ADT, biopsy

## Abstract

**Simple Summary:**

In this paper, we look at the role of artificial intelligence (AI) advancements in prostate cancer diagnosis and management. Specifically, we focus on magnetic resonance prostate reconstruction, prostate cancer detection/stratification/reconstruction, positron emission tomography/computed tomography, androgen deprivation therapy, and prostate biopsy. A total of 64 studies were included. Our results showed that deep learning AI models in prostate cancer diagnosis show promise but are not yet ready for clinical use due to variability in methods, labels, and evaluation criteria. Conducting additional research while acknowledging the limitations is crucial for reinforcing the utility and effectiveness of AI-based models in clinical settings.

**Abstract:**

Background: The aim was to analyze the current state of deep learning (DL)-based prostate cancer (PCa) diagnosis with a focus on magnetic resonance (MR) prostate reconstruction; PCa detection/stratification/reconstruction; positron emission tomography/computed tomography (PET/CT); androgen deprivation therapy (ADT); prostate biopsy; associated challenges and their clinical implications. Methods: A search of the PubMed database was conducted based on the inclusion and exclusion criteria for the use of DL methods within the abovementioned areas. Results: A total of 784 articles were found, of which, 64 were included. Reconstruction of the prostate, the detection and stratification of prostate cancer, the reconstruction of prostate cancer, and diagnosis on PET/CT, ADT, and biopsy were analyzed in 21, 22, 6, 7, 2, and 6 studies, respectively. Among studies describing DL use for MR-based purposes, datasets with magnetic field power of 3 T, 1.5 T, and 3/1.5 T were used in 18/19/5, 0/1/0, and 3/2/1 studies, respectively, of 6/7 studies analyzing DL for PET/CT diagnosis which used data from a single institution. Among the radiotracers, [^68^Ga]Ga-PSMA-11, [^18^F]DCFPyl, and [^18^F]PSMA-1007 were used in 5, 1, and 1 study, respectively. Only two studies that analyzed DL in the context of DT met the inclusion criteria. Both were performed with a single-institution dataset with only manual labeling of training data. Three studies, each analyzing DL for prostate biopsy, were performed with single- and multi-institutional datasets. TeUS, TRUS, and MRI were used as input modalities in two, three, and one study, respectively. Conclusion: DL models in prostate cancer diagnosis show promise but are not yet ready for clinical use due to variability in methods, labels, and evaluation criteria. Conducting additional research while acknowledging all the limitations outlined is crucial for reinforcing the utility and effectiveness of DL-based models in clinical settings.

## 1. Introduction

Prostate cancer (PCa) is the second most frequent cancer in men and the fifth leading cause of death worldwide [1]. This has prompted the rapid development of methods for diagnosing and treating PCa. Thus, modern technology allows for the thorough examination of the pelvis using multiparametric magnetic resonance imaging (mpMRI) of the whole body using positron emission tomography (PET/CT) to better identify damage to regional lymph nodes and distant metastases of the prostate to determine tumor aggressiveness, and androgen deprivation therapy (ADT) has been used as the first treatment method for high-risk patients [2]. However, the implementation and interpretation of the abovementioned studies depend on the subjective opinion of a specialist, which can be subject to deviations for various reasons. Moreover, the manual layer-by-layer segmentation of images is time-consuming and subject to the experience of specialists. This leads to differences in opinions among specialists, unnecessary biopsies of indolent neoplasms, and the active monitoring of patients with clinically significant forms [3]. Researchers have developed automated methods using artificial intelligence (AI) techniques to address these challenges. These methods can reduce the time required for MRI, PET/CT, ultrasound (US), and digital pathology interpretation and improve the accuracy and consistency of PCa diagnosis [4]. Moreover, such second-opinion systems allow for the better selection of treatment methods, such as ADT. Deep learning (DL), a branch of machine learning (ML), can automatically learn and extract hierarchical representations of data, making it adept at handling complex and unstructured data and improving accuracy over time, unlike traditional machine-learning models that require significant manual feature engineering. While DL algorithms show promise in PCa diagnosis, their success naturally depends on different variables, such as dataset type, imaging protocol variability, human participation during training, and proper validation and testing [5].

This review aimed to analyze the current state of DL-based PCa diagnosis with a focus on prostate reconstruction, PCa detection/stratification/reconstruction, PSMA-PET diagnosis, and the use of DL in the context of ADT and prostate biopsy, associated challenges, and their clinical implications.

## 2. Methods

In October 2023, a search was conducted in the PubMed and Google scholar databases via Boolean operators with the use of the following terms: “Prostate”, “cancer”, “PCa”, “MRI”, “PET/CT”, “ADT”, “biopsy”, “reconstruction”, “detection”, “stratification”, “AI”, “deep learning”, “DL”. The inclusion criteria were the availability of a full article in English; a publication date no more than five years from the search date; the use of DL methods for MR-based PCa diagnosis and prostate reconstruction; PET/CT, ADT, and prostate biopsy assistance; a description of the datasets used (including the number of institutional and open datasets and the number of cases); a description of the segmentation of the area of interest when training a neural network (manual, semi-autonomous, or autonomous); and the presence of a description of validation or testing (internal or external). In addition, the references in the selected articles were analyzed. If an article compared various algorithms, the data on the most efficient algorithm were entered into the table.

Descriptive statistics were performed using the SPSS Statistics software (version 26.0). The distribution of continuous data was determined using the Kolmogorov–Smirnov test. Depending on the normalization, the mean value with standard deviation or the median with minimum and maximum values was calculated.

## 3. Results

**Figure 1:**
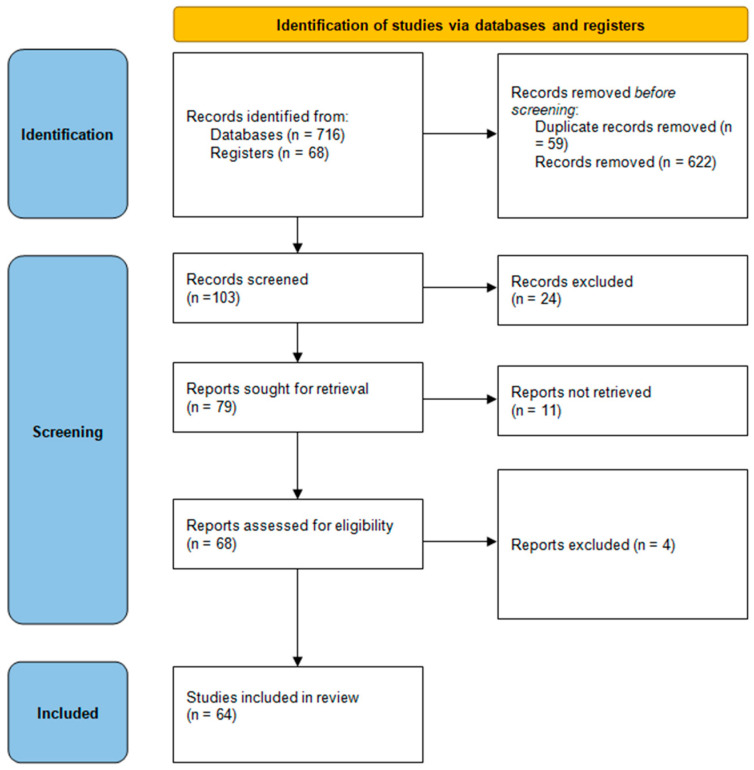
PRISMA search strategy for selection of related and relevant research.

As a result of this search, 784 articles were identified, of which, 64 were included (Figure 1). 

Prostate reconstruction was performed in 21 studies. The reconstruction of prostate cancer has been described in six studies. The detection and stratification of prostate cancer has been described in 22 articles. PET/CT was performed in seven studies. ADT and prostate biopsy optimization were analyzed in two and six studies, respectively.

### 3.1. MR-Based Prostate Reconstruction

**Figure 2:**
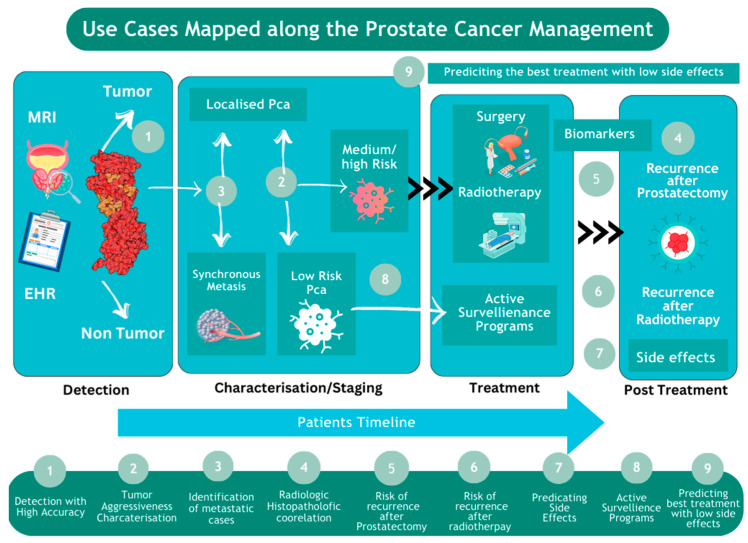
The use of AI for prostate cancer across patient timeline.

A summary of previous studies describing deep learning models for 3D prostate reconstruction is presented in Table 1/Figure 2. Da Silva et al. [6] proposed an automatic and novel coarse-to-fine segmentation method for prostate 3D MRI scans. The coarse segmentation step combines local texture and spatial information using the Intrinsic Manifold Simple Linear Iterative Clustering algorithm and a probabilistic atlas in a deep convolutional neural network (DCNN) model jointly with the particle swarm optimization algorithm to classify prostate and non-prostate tissues. Then, fine segmentation uses the 3D Chan–Vese active contour model to obtain the final prostate surface. This approach led to a DSC value of 0.85. Wang et al. presented a three-dimensional (3D) fully convolutional network (FCN) with deep supervision and group dilated convolution [7]. According to the results, the proposed method leads to a DSC of 0.86 and significantly prevails over UNet (0.836, *p* = 0.023) and VNet (0.838, *p* = 0.018). 

Liu et al. proposed a novel Densely Dilated Spatial Pooling Convolutional Network (DDSP ConNet) in an encoder–decoder structure [8]. It employs a dense structure to combine dilated convolution and global pooling, thus supplying coarse segmentation results from the encoder and decoder subnets and preserving more contextual information. Nai et al. evaluated the effectiveness of monomodal DenseVNet, multimodal ScaleNet, and mono- and multimodal HighRes3DNet [9], and the DSC in whole-prostate reconstruction for these networks was 0.875, 0.848, 0.858, and 0.890, respectively. Multimodal HighRes3DNet and ScaleNet had a higher DSC, with a statistically significant difference only in the zonal reconstruction of the peripheral zone and the central part of the prostate compared to monomodal DenseVNet, which indicates an increase in the accuracy of regional segmentation when using multimodal networks. However, the difference was statistically insignificant in terms of isolating the entire prostate. Yu et al. [10] proposed a novel volumetric convolutional neural network (ConvNet) with mixed residual connections to handle variations in prostate shape and indistinct prostate boundaries. Karimi et al. described the architecture of a convolutional neural network and a training strategy that aimed to exploit the limited variability in the prostate shape while simultaneously solving the problem of insufficient data for training [11]. The key to achieving these goals is a statistical shape model. The output of the neural network is limited by the parameters allowed by the shape model. Ushinsky et al. described the use of a 3D/2D hybrid neural network, UNet, which achieved a DSC value of 0.898. A hybrid 3D/2D U-network was created by modifying the down-sampling portion of the U-network to perform convolution image processing, ReLU activation, and normalization in 3D [12]. These 3D images are then mapped using a projection operation to match the 2D images in the U-Net upsampling step. Yan et al. described the application of a neural network with a backpropagation algorithm consisting of three components, convolution and pooling layers (CPlayers), a propagation layer (P-layer), and an F-measure loss layer (L-layer), and compared this approach with FCN-16s and FCN-32s, DS-Net, and VolConv [13]. The DSC values for the described and compared networks were 0.9055, 0.8658, 0.6983, 0.6693, and 0.904, respectively. Jia et al. described a 3D Adversarial Pyramid Anisotropic Convolutional Network (3D APA-Net) consisting of a generator (i.e., 3D PA-Net) that performs image segmentation and a discriminator (i.e., a six-layer convolutional neural network) that distinguishes between the segmentation result and its corresponding ground truth. The 3D PA-Net has an encoder–decoder architecture that consists of a 3D ResNet encoder, anisotropic convolutional decoder, and multilayer pyramidal convolutional connections [14]. The resulting DSC values were compared to those of VNet, 3D GCN, and 3D UNet and were 0.901, 0.796, 0.817, and 0.818, respectively. Comelli et al. compared the accuracy of prostate reconstruction using U-Net, E-Net, and ERFNet [15]. According to the results, the first two networks provided a DSC of over 0.9, whereas the best accuracy was achieved when using ENet (0.9089). Bardis et al. described the sequential use of three convolutional neural networks, each of which was implemented using an individual hybrid 3D/2D U-Net architecture [16]. The networks were named U-NetA, U-NetB, and U-NetC, respectively. U-NetA is responsible for localizing the prostate. U-NetB is responsible for prostate segmentation, whereas zonal segmentation is implemented using U-NetC. The DSC values for the entire prostate, transitional zone, and peripheral zone are 0.940, 0.910, and 0.774, respectively. Sanford et al. focused on the problem of heterogeneity in MR images taken from different sources and described the use of 2D–3D anisotropic hybrid networks and deep multilevel transformation as a data-augmentation method [17]. This approach provided segmentation accuracy for the entire prostate and transition zone, with values of 0.915 and 0.897, respectively. Liu et al. described the use of a neural network using batch normalization and a loss function to level the differences between MR images of the prostate obtained using different MR scanners [18]. Thus, the authors obtained a DSC value of 0.9169. Wang et al. described a conditional generative adversarial network (SegDGAN). The generator G is a segmentation network in which end-to-end learning is performed. While G uses a U-network encoder–decoder structure [19], D is a multidimensional feature extraction network with six layers. Each layer contains a convolution layer, BN layer, and rectified linear unit activation function layer. The highest DSC value was 0.9166, which was significantly higher than the segmentation accuracy using U-Net, FCN, and SegAN. Aldoj et al. developed a Dense U-net algorithm based on the previously tested DenseNet and U-net networks. Compared to U-net, the Dense U-net achieved an average Dice score in whole-prostate segmentation of 0.921 vs. 0.907, for the central part (central + transition zone), of 0.895 vs. 0.891, and for the peripheral zone, of 0.781 vs. 0.75 [20]. To et al. described a 3D deep dense convolutional neural network and compared its accuracy in prostate segmentation with 3D U-Net, 2D DS-net, and 3D MRC-net. The DSC values were 0.9511, 0.9380, 0.9247, and 0.9237, respectively [21]. Zhu et al. described a boundary-weighted adaptive neural network (BOWDA-Net) with a DSC value of 0.9254 for automated prostate segmentation [22]. Zhu et al. described the use of two cascade U-Nets: the first determined the contour of the entire prostate and the second was responsible for the segmentation of the peripheral zone. The DSC values were 0.927 and 0.793, respectively, which were significantly higher than those obtained using a single UNet network [23]. Meyer et al. used an anisotropic 3D multi-stream CNN with an encoder–decoder architecture with four levels of resolution, similar to the 3D U-Net. Reconstruction was performed using multiplanar T2 images and compared to the monoplanar approach. A statistically significant increase in DSC was observed at all prostate levels, especially at the base (0.906 vs. 0.898) and apex (0.901 vs. 0.888). The total DSC level for the entire prostate was 0.933 [24]. Chen et al. described an AlexNet network using batch normalization and global maximum pooling algorithms, which achieved a DSC value of 0.921 [25]. Yan et al. described the use of the Pyramid Scene Parsing Network (PSPNet) and compared its accuracy with that of FCN and Unet [26]. The DSC values for PSPNet, FCN, and U-net were 0.985, 0.8924, and 0.9107, respectively.

### 3.2. MR-Based PCa Detection and Stratification

Studies on PCa detection and stratification via DL algorithms are listed in Table 2. Ishioka et al. used a CNN architecture that combined U-net with ResNet50. U-net is responsible for distinguishing whole and local pelvic structures and is capable of differentiating cancerous regions from other structures [27]. ResNet reformulates the layers as learning residual functions with reference to the layer inputs, instead of learning unreferenced functions. The authors reported AUC values of 0.645 and 0.636 for the two validation datasets. Zabihollahy et al. described a model made up of three U-Nets (ADC-Net1, ADC-Net2, and ADC-Net3) with different capacities and weights to effectively learn the characteristics of PCa in the PZ from ADC maps calculated from DWI with diffusion factors of 0, 500, and 1000 s/mm^2^, respectively. The resulting AUC, sensitivity, and specificity for the combined network were 0.779, 85.76%, and 76.44% [28]. Mehrtash et al. implemented a 3D-CNN with three inputs: ADC, DWI at the maximum value of the diffusion factor, and Ktrans. The performance of different combinations of mpMRI inputs to the CNN was assessed, and the best result was achieved using DWI and DCE-MRI modalities with an AUC equal to 0.80 [29]. Saha et al. described an architecture consisting of two parallel CNNs (M1 and M2). M1 was used to generate the preliminary voxel-level detection of csPCa in prostate bpMRI scans, whereas the goal of the M2 classification network was to improve the overall model specificity via the independent, binary classification of each scan and its constituent segments. According to the results, the described architecture, when using both networks, reached an average of 0.885 AUC for PCa identification, which was significantly higher than when using networks such as Attention U-Net, nnU-Net, UNet++, and U-SEResNet (0.861, 0.872, 0.850, 0.856, and 0.500, respectively) [30]. Chen et al. described transfer deep learning using InceptionV3 and VGG-16 networks for PCa detection using T2, DWI, and DCE images. The transfer learning method involves adapting a network designed for a related task. According to the authors’ results, the InceptionV3 model achieved an AUC of 0.81 and VGG-16 reached 0.83 [31]. Sobecki et al. proposed a model that was built upon the VGG-16 core network to a 3D model by introducing 3D convolutional layers instead of 2D layers. T2 images in the sagittal, coronal, and axial planes, as well as DWI, ADC, and DCE images, were independently processed in separate 3D layers. The optimized model with knowledge encoding on training achieved several better classification results than the traditional architecture (AUC of 0.84 vs. AUC of 0.82) [32]. Sanyal et al. described a two-stage convolutional neural network model of two U-Net networks. The first is responsible for the segmentation of the prostate gland, followed by the detection of clinically significant prostate cancer. The described architecture achieved an AUC of 0.86 [33]. Bhattacharya et al. proposed CorrSigNet, an automated two-step model that localizes prostate cancer on MRI by capturing the pathological features of the cancer [34]. First, the model learns the MRI signatures of cancer that are correlated with the corresponding histopathological features using Common Representation Learning. Second, the model uses the learned correlated MRI features to train a convolutional neural network to localize prostate cancer. The final AUC was 0.86. Yu et al. described Res-UNet. The detection network had a UNet architecture with 2D residual blocks. Res-UNet was designed to have five down-sampling and five up-sampling residual blocks [35]. Yoo et al. proposed a modified ResNet. Five networks were used to analyze the ADC images with the appropriate diffusion factors (0, 100, 400, 1000, and 1600 s/mm^2^). To improve the performance of the architecture, the authors implemented a fully preactivated residual network. In the original ResNet, batch normalization and ReLU activation layers were followed after the convolution layer, but in the pre-activation ResNet, batch normalization and ReLU activation layers came before the convolution layers [36]. Zhong et al. used the ResNet neural network with transfer learning to distinguish between clinically insignificant and significant PCa foci and compared its effectiveness with a standard convolutional neural network and PI-RADS v2 conclusion. According to the results, the AUC for distinguishing indolent from clinically significant lesions using the transfer learning model, without it, and the PI-RADS v2 scores were 0.726, 0.687, and 0.711, respectively; however, the difference between transfer learning and PI-RADS v2 was not statistically significant [37]. Khosravi et al. used Google Inception-V128 (GoogLeNet) architecture. The authors pursued two goals: to differentiate tumors from healthy tissues by grouping ISUP groups (IG) 3, 4, and 5, and to identify only clinically significant lesions (by grouping IG 1 and 2 and 3, 4, and 5 into different groups). The proposed algorithm was able to identify PCa and, in particular, isolate clinically significant lesions with an AUC, sensitivity, and specificity of 0.89, 81.5%, and 82% and 0.78, 71.3%, and 68.9%, respectively [38]. Arif et al. described the experience of using deep learning on T2, DWI (b-factor of 800 s/mm^2^), and ADC counterparts in the detection of clinically significant prostate cancer and in the confirmation of low-risk cancer (ISUP grade ≤ 1) in patients under active surveillance. Two MRI sequences (T2w and DWI) and an ADC map were used as separate input channels for the model. AUC, sensitivity, and specificity ranged from 0.65 to 0.89, 82 to 92%, and 43 to 76%, respectively [39]. Wang et al. described the use of a neural network consisting of pooled subnets: (1) a tissue deformation network (TDN) for automated prostate detection and multimodal registration and (2) a dual-path convolutional neural network (CNN) for clinically significant (CS) PCa detection. During the training phase, the two networks mutually influence each other and effectively guide the registration and extraction of the representative features. According to the results of the authors, the combination of T2 and ADC images is justified and allows an AUC of 0.8978 [40]. Abdelmaksoud et al. assessed the diagnostic accuracy of AlexNet and VGGNet networks using ADC calculated from DWIs with nine diffusion factors (100, 200, 300, 400, 500, 600, 700, 800, and 900 s/mm^2^). The mean sensitivity and specificity of AlexNet were 87.5 ± 2.3% and 90.9 ± 1.9%, respectively. These results improved when using a deeper CNN model (VGGNet), reaching values of 91.7 ± 1.7% and 90.1 ± 2.8%, respectively [41]. Aldoj et al. tested a 3D convolutional neural network with separate input layers for the T2, DWI, ADC, and DCE sequences. The AUC value reached 0.91 using DWI, ADC, and DCE, whereas the use of T2 did not lead to any improvement [42]. Song et al. modified VGGNet and developed a deep convolutional neural network (DCNN) architecture. Training was performed using T2, DWI, and ADC images from 195 patients. The AUC for distinguishing between tumor and healthy tissues was 0.944, with a sensitivity of 87.0% and a specificity of 90.6%. Decision curve analysis revealed that the joint model of PI-RADS v2 and DCNN provided additional net benefits compared with the DCNN model and PI-RADS v2 alone [43]. Pellicer-Valero et al. tested a 3D Retina U-Net for prostate tumor detection and stratification by combining a single-stage RetinaNet and U-Net. The network achieved an AUC, sensitivity, and specificity of 0.96, 100%, and 79%, respectively [44]. Xu et al. presented a deep learning framework using ResNets to identify suspicious lesions on prostate mp-MRIs. The residual network achieved an AUC of 97 for lesion detection, with an average Jaccard score of 71%, which compared the agreement between network and radiologist segmentation [45]. Cao et al. described the use of FocalNet, an end-to-end multiclass CNN that simultaneously determines the lesion and its grade of malignancy according to ISUP grading by accepting T2 and ADC images into two input channels and predicting six classes of labels at the pixel level: no lesion, ISUP 1, 2, 3, 4, and 5. To detect only clinically significant lesions, an area under the characteristic curve of 0.81 was achieved [46]. Hou et al. described ResNeXt, which is a 2D convolutional neural network with a convolutional block attention module (CBAM) for analyzing mpMRT images by combining T2, DWIs with a diffusion factor of 1500 s/mm^2^, and ADC. The model using a single-slice image yielded the highest areas under the receiver operating characteristic curve (AUC) of 0.857 (95% confidence interval [CI], 0.827–0.884), 0.807 (95% CI, 0.735–0.867), and 0.728 (95% CI, 0.631–0.811) in the training, validation, and test data, respectively. The performance of the two experts (AUC, 0.632–0.741 vs. 0.715–0.857) was lower (paired comparison, all *p* values < 0.05) than that of AI assessment [47]. Zong et al. utilized a vanilla CNN of the VGG style that consists of four convolutional layers, each followed by batch normalization and ReLU nonlinear layers, and two max pooling layers after every two convolutional blocks. The maximum values of AUC, sensitivity, and specificity were 0.91, 100%, and 83%, respectively, with the combination of T2, DWI, and ADC sequences [48].

### 3.3. MR-Based Prostate Cancer Reconstruction

Studies on PCa 3D reconstruction are presented in Table 3.

Gunashekar et al. used a 3D convolutional neural network for PCa segmentation based on a U-Net network. To interpret the CNN segmentation results, heat maps were generated using a gradient-weighted class activation map (Grad-CAM). DSC was 0.32 and did not significantly differ from the results of manual tumor annotation by radiologists [49]. De Vente et al. used a 2D U-Net with MRI slices as the input and lesion segmentation maps that encoded the ISUP grade, a measure of cancer aggressiveness, as the output. The model scored a voxel-wise weighted kappa of 0.446 ± 0.082 and a Dice similarity coefficient for segmenting clinically significant cancers of 0.370 ± 0.046, obtained using 5-fold cross-validation [50]. Lai et al. used a DCNN model called SegNet, which has an encoder–decoder structure, to reconstruct PCa via T2, DWI, and ADC sequences as well as to determine the most informative combination. According to the results, all sequences used combinations that led to the best DSC of 0.5273 [51]. Lee et al. described two combinations of the U-Net network and a convolutional GRU, UconvGRU and SUconvGRU, which allow the modeling of both spatial and temporal patterns. DSC values of 0.2164 and 0.5323 were obtained [52].

Chen et al. proposed a multibranch UNet (MB-UNet) for PCa reconstruction based on T2, DWI (*b*-value > 1000 s/mm^2^) and ADC maps. The DSC of the test sample reached a value of 0.6333. The authors emphasized the importance of the DWI sequence for PCa segmentation [53]. Alkadi et al. described their deep learning architecture with an encoder, a corresponding decoder of the same size, a SoftMax trainable layer, and a pixel-classification layer. This technique was tested for the segmentation of prostate cancer using T2 images and reached a DSC of 0.892 [54].

### 3.4. Positron Emission Tomography (PET)

Studies on the use of DL with PET in patients with PCa are summarized in Table 4.

Hartenstein et al. [55] tested whether convolutional neural networks (CNNs) could accurately predict the ^68^Ga-PSMA-PET/CT lymph node status using only CT images. Experienced radiologists had an AUC of 0.81, while convolutional neural networks (CNNs) had 0.95 (a status) and 0.86 (balanced location, masked). CNNs improved their effectiveness by “learning” infiltration probabilities in different anatomical locations. Capobianco et al. [56] used training data from two radiotracers to develop a deep-learning method for stage-based image categorization of the PET/CT of ^68^Ga-PSMA-11. Training strategies were utilized to maximize data use from more ^18^F-FDG PET/CT images and expert annotations. This comprised transfer learning and combination training with tracer-type inputs to the network. Following the PROMISE miTNM architecture, we assessed network and expert annotations for N- and M-stage concordance. Training with ^18^F-FDG data and the development set yielded an average precision of 80.4% (confidence interval: 71.1–87.8) for suspect uptake sites compared to expert judgment. It classified anatomical locations of suspicious findings with 77% (CI: 70.0–83.4) accuracy. In addition, 81% agreed on regional lymph node involvement and 77% agreed on metastatic stage. Experts and the algorithm agreed on the anatomical location of problematic uptake regions in whole-body ^68^Ga-PSMA-11 PET/CT. PSMA–ligand data are scarce; therefore, training samples from another radiotracer improves the performance. To automatically segment intraprostatic cancer lesions on PSMA PET scans, Ghezzo et al. [57] tested a cutting-edge convolutional neural network on a diverse cohort. Compared to hand contouring, the AI model performed relatively well, with a median Dice score of 0.74. Kendrick et al. [58] developed and tested a computerized method that uses advanced ML to identify and distinguish metastatic prostate cancer (mPCa) lesions in whole-body [^68^Ga]Ga-PSMA-11 PET/CT images. The goal of this approach was to extract patient-level prognostic indicators. The accuracy, sensitivity, and positive predictive value (PPV) for each patient were all greater than 90%, with the best at 97.2%. The lesion-level PPV and sensitivity were 88.2% and 73.0%, respectively. Inter-observer variability was examined using the Dice similarity coefficient (DSC) and PPV at the voxel level. The median DSC was 50.7% for the first observer and 32% for the second observer, with a *p*-value of 0.012. The median PPV was 64.9% for the first observer and 25.7% for the second observer (*p* < 0.005). Kaplan–Meier analysis showed a strong correlation between TLVauto and TLUauto and patient survival (both *p* < 0.005). Using PSMA PET scans of patients with PCa, Leung et al. [59] developed a DL and radiomics system to categorize lesions and patients. Based on the PSMA PET scans of 267 male patients with PCa, 3794 lesions were grouped into the PSMA-RADS groups. The framework had lesion-level and patient-level PSMA-RADS classification AUROC scores of 0.87 and 0.90 on the test set. In the test set, the framework had lesion- and patient-level prostate cancer classification AUROC values of 0.92 and 0.85. Trägårdh et al. [60] developed an AI-driven method to detect and measure prostate tumors, lymph nodes, and bone metastases using [^18^F] PSMA-1007 PET/CT images. AI segmentation has been compared with the hand segmentations of numerous nuclear medicine doctors. The AI technique had 79% sensitivity for prostate tumor/recurrence, 79% for lymph node metastasis, and 62% for bone metastasis. The sensitivity of nuclear medicine physicians averaged 78%, 78%, and 59%, respectively. Zhao et al. [61] used a deep neural network to automatically characterize prostate cancer (PC) lesions. This approach evaluates the tumor burden on ^68^Ga-PSMA-11 PET/CT scans to optimize the PSMA-directed radionuclide therapy. ^68^Ga-PSMA-11 PET/CT imaging was performed in 193 patients with mPCa at three medical facilities. Scientists have focused on pelvic bone and lymph node lesions for their proof-of-concept. The triple-combining 2.5D U-Net neural network automatically classified these irregularities. The recommended method collects axial, coronal, and sagittal plane information simultaneously, mimicking the clinician’s workflow and reducing computational and memory requirements. The network detected bone lesions with 99% accuracy, 99% recall, and a 99% F1 score. The network detected lymph node lesions with 94% precision, 89% recall, and a 92% F1 score.

### 3.5. Androgen Deprivation Therapy (ADT)

Studies on the use of DL with ADT for patients with PCa are summarized in Table 5.

Spratt et al. [62] used digital pathology images and clinical data from the pretreatment prostate tissue of 5727 individuals in five phase III randomized studies who received radiation with or without ADT. An AI-based predictive model was created and validated to assess ADT’s ability to prevent distant metastasis, which is the key outcome measure. After model completion, NRG/RTOG 9408 was validated by randomizing 1594 males to either radiation alone or radiotherapy with ADT for 4 months. The prediction model showed that ADT significantly reduced distant metastasis in positive patients (*n* = 543, 34%) compared with radiotherapy alone. With a 95% CI of [0.19–0.63], the hazard ratio (sHR) was 0.34 (*p* < 0.001). No significant differences were observed between the treatment groups in the prediction model-negative subgroup of 1051 patients (66%). The subdistribution hazard ratio (sHR) was 0.92, with a 95% confidence interval of 0.59–1.43 (*p* = 0.71). Mobadersany et al. [63] developed a novel AI technique that uses clinical, digitized H&E, and radiology bone scan (rBS) data to predict outcomes in non-metastatic castration-resistant prostate cancer (nmCRPC) patients who received ADT. By integrating imaging data with 11 conventional clinical features (such as tumor stage, ISUP grade, PSA), the authors developed and taught a multimodal technique that uses survival convolutional neural networks (SCNNs) and the Cox proportional-hazards model (CPH) to assess ADT outcomes for overall survival (OS) and time to PSA progression (TTP). The trained framework was tested for risk stratification and prediction using the hold-out set. Bootstrapping analysis with the Wilcoxon signed-rank test determined the multimodal framework’s performance advantage over clinical CPH. ADT for overall survival (OS) and time to progression (TTP) in nmCRPC was reliably predicted using a multimodal approach. The multimodal approach in SPARTAN’s hold-out set improved clinical CPH prediction by 14–16% across both outcomes. The Wilcoxon signed-rank test with a *p*-value < 0.0001 showed that this improvement was significant.

### 3.6. Prostate Biopsy

Studies on the use of DL to assist prostate biopsies are summarized in Table 6.

Sedghi et al. [64] used transrectal ultrasonography (TeUS) data and a deep neural mapping (DNM) model to accurately map PCa distribution in an unsupervised manner. The TeUS data are transformed into a topological hyperlattice with related samples closer together. Thus, malignant prostate and noncancerous tissues are categorized by similarity. The UroNav device, invented by Florida-based In vivo Corporation, merges MRI and ultrasound images during guided biopsy. The ultrasound transducer was held steady for 5 s to acquire 100 TeUS frames before initiating the biopsy gun. A tissue sample was then taken using a biopsy needle. Strong consensus cores achieved a projection AUC of >0.8.

Azizi et al. [65] demonstrated a consistent software architecture that processes TeUS data using recurrent neural networks. A comprehensive clinical trial of 157 individuals and 255 biopsy cores used ultrasound data to evaluate the accuracy of cancer detection. Additionally, 21 biopsy targets from six participants were used for testing. The authors obtained an AUC of 0.85. 

Van Sloun et al. [66] used DL and U-net architecture to separate the prostate (zone) automatically and in real time using TRUS images from multiple scanners. The pixel accuracy, Jaccard index, and Hausdorff distance were used to evaluate the zonal segmentation. The traditional automated prostate segmentation algorithm was significantly inferior to the sophisticated DL method. It had a median accuracy of 98%, a Jaccard index of 0.93 (range: 0.80–0.96), and a Hausdorff distance of 3.0 mm. Zonal segmentation achieved pixel-wise accuracy of 97% (95–99%) for the perimeter zone and 98% (96–99%) for the transition zone. A supervised DL system by Orlando et al. [67] correctly delineated the prostate in 3D TRUS images from several facilities using diverse acquisition techniques and US machine models. An adaptable algorithm for needle-based PCa procedures is required. A 2D U-net model was compared to 3D reconstruction and optimized 3D networks, such as 3D V-Net, Dense V-Net, and high-resolution 3D-Net. This study examines how 2D picture predictions may lose spatial and structural information. The proposed design had DSC, recall, precision, VPD, MSD, and HD of 94.1%, 96.0%, 93.2%, 5.78%, 0.89 mm, and 2.89 mm, respectively. In almost all measurements, the proposed technique outperformed the top-performing optimized 3D network, that is, 3D V-Net with a Dice plus cross-entropy loss function. The average prostate segmentation time was less than 0.7 s, which is suitable for surgery. To et al. [68] introduced LensePro, which is a two-stage system. The first stage involves self-supervised learning to derive good feature representations from unlabeled TRUS data. In the second step, the generated features are classified using prototype-based learning, which tolerates label noise. Based on 124 systematic prostate biopsy patients, LensePro diagnosed prostate cancer (PCa) on ultrasound with an AUROC of 77.9%, sensitivity of 85.9%, and specificity of 57.5%. A powerful deep learning algorithm developed by Soerensen et al. [69] efficiently and precisely separated the prostate on MRI scans. This model was effectively integrated into a clinical magnetic resonance-ultrasound fusion biopsy. Prospectively, the authors integrated ProGNet architecture into fusion biopsy for 11 patients. It was also tested against the U-Net, holistically nested edge detection, and radiology technicians. ProGNet outperformed U-Net, a holistically nested edge detector, and radiology technicians in the retrospective internal test set with a Dice similarity coefficient (DSC) of 0.92 (*p* < 0.0001). A prospective cohort study found that ProGNet outperformed radiology technicians (DSC = 0.93, *p* < 0.0001) (DSC = 0.90). ProGNet created a clinically viable segmentation file for 35 s for each case, compared with 10 min for radiology technicians.

## 4. Discussion

**Figure 3:**
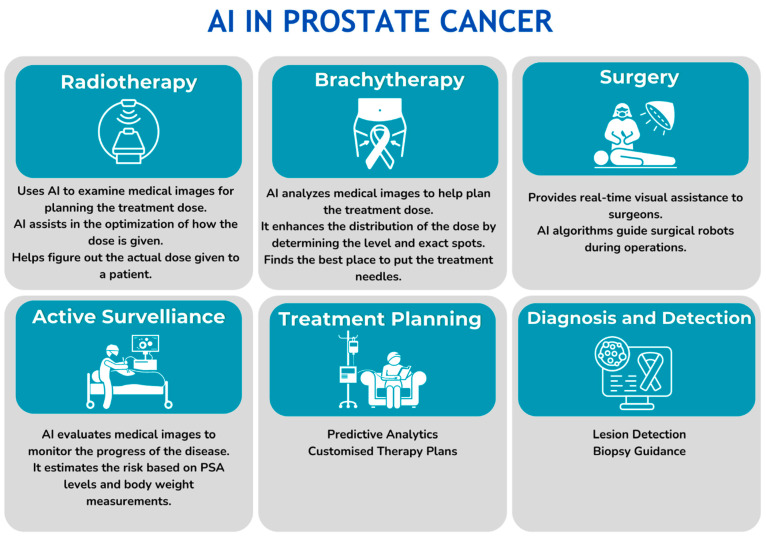
Role of AI in all aspects of prostate cancer diagnosis and treatment.

AI can aid in all aspects of prostate cancer diagnosis and treatment selection (Figure 3). Despite the anatomical and functional visualization of the prostate and whole body when performing mpMRI and PET/CT, the problems of the need for manual prostate contouring for counseling of the area of interest, inter-observer variability in examination, the performance of unnecessary biopsies, and omission of the dominant and most malignant foci lead to the incorrect stratification of patients. Moreover, when performing a biopsy, the problem of up- and down-grading remains, which emphasizes the importance of developing various second-opinion systems both at the stage of diagnosing PCa and when performing a biopsy [70,71].

One of the ways to solve these problems is the use of artificial intelligence, particularly deep learning methods [72]. As a result of the search, we selected 64 papers describing deep learning methods to alleviate prostate and PCa visualization in prostate reconstruction, as well as the detection, stratification, and reconstruction of prostate cancer, which provided detailed information regarding the data used, their processing, the development of the neural network, and its approximation. Despite the impressive results that emphasize the promise and relevance of such supporting systems, the final efficiency and generalization of neural networks depends not only on the novelty of the network itself but also on many other variables [73]. Youn et al. compared the accuracy of the Prostate AI system (Siemens Healthcare, Tokyo, Japan), with the results of radiologists divided into subgroups depending on their experience in mpMRI interpretation. Only the opinion of the experts with the least experience was inferior to the assessment of Prostate AI, while the most experienced radiologists achieved significantly greater accuracy [74]. 

The initial obstacle in confirming the generalizability of such DL-based models is the data quality used for training. If the training data are limited in diversity, the resulting model may not perform well on data from different centers or acquired using different protocols. This is more obvious from a texture analysis point of view. Castillo et al. evaluated the generalizability of radiomics models for prostate cancer classification [75]. The three single-center models obtained a mean AUC of 0.75, which decreased to 0.54 when the model was applied to the external data. In a multi-center setting, the radiomics model obtained a mean AUC of 0.75. Among the papers analyzing MR-based prostate reconstruction and PCa diagnosis, a multi-center dataset (two or more, regardless of its institutional or open nature) was combined in 15 studies (31%), and none of these investigated PCa reconstructions. A single magnetic field power of 3 T and 1.5 T was observed in 42 (86%) and 1 (2%) paper, respectively, whereas the dataset with combined magnetic field power was used in only 6 (12%) studies. Moreover, multivendor images (the use of a dataset with images from two or more MRI scans) were used in 38 (78%) cases. The same problem was observed in other clinical scenarios. So, six of the seven studies on DL implementation in PET/CT were also performed using a single-center dataset.

Similar to the data diversity, the number of cases used also influences the accuracy of the created models. According to Hosseinzadeh et al., PI-RADS-trained DL can accurately detect and localize ISUP > 1 lesions but can achieve expert performance using substantially more than 2000 training cases [76]. Among the studies investigated in our review, the minimum case numbers for prostate reconstruction, PCa detection/stratification, PCa reconstruction, PET/CT, ADT, and biopsy were 25, 37, 16, 34, 154, and 157, respectively, making such studies in the prototype stage and restricting their results’ arguability in favor of DL-based systems [77].

The next drawback of the papers analyzed is the limited exploration of the potential impact of sequence selection on the performance of DL algorithms, which is particularly obvious among studies on MRI diagnosis. Only a few studies have attempted to estimate the added value of different sequences, instead of using those chosen in advance. Aldoj et al. [42] stated that the use of T2 did not lead to any improvement in PCa detection accuracy, whereas Wang et al. [40] indicated that the combination of T2 and ADC images was justified for this purpose. According to Mehrtash et al. [29], the best accuracy of 3D-CNN for detecting PCa was achieved with DWI at the maximum value of the diffusion factor and Ktrans, whereas ADC use was not profitable. However, a recent meta-analysis revealed that among all mpMRI sequences, ADC correlated significantly with the ISUP grade [78]. Not in the focus of DL but related to AI-based methods generally, Bonekamp et al. compared biparametric contrast-free radiomic machine learning (RML) and the mean apparent diffusion coefficient (ADC) for the characterization of prostate lesions detected during prospective MRI interpretation. According to the results, radiomics had comparable but not better performance than the mean ADC assessment [79].

The nature of prostate cancer determines the complexity of using DL for characterization on MRI images. In all the studies analyzed, only a dominant focus was found, which did not allow the determination of the true degree of PCa spread [80]. In addition, although in all studies within PCa detection/stratification and PCa reconstruction groups, the main task was to reveal a tumor within the entire prostate, not all studies either declared PCa distribution in different prostatic zones within dataset cases or used cases with all possible scenarios of PCa location. Among the 28 papers, only 18 (64%) indicated PCa locations in the dataset. Cases with PCa only in the PZ (peripheral zone), PZ + TZ (Tz—transitional zone), PZ + TZ + CZ (CZ—central zone), PZ + TZ + AS + SV (AS—anterior stroma; ZV—seminal vesicle) zones were used in 2, 5, 1, and 10 studies, respectively. Unfortunately, almost half of these studies did not provide this information. However, these mostly used open datasets (particularly ProstateX, combining cases with PCa from PZ + TZ + AS + SV) instead of including institutional ones to make the final dataset more heterogeneous. 

The segmentation approach during training is also crucial and is one of the steps most susceptible to bias. According to Bleker et al., a deep learning mask (DLM) auto-fixed VOI placement is more accurate in detecting CS PCa and can result in a 97% reduction in time [81]. Manual segmentation is a labor-intensive and time-consuming process that makes scaling up to large datasets difficult. Moreover, there can be significant differences in segmentation between different human observers, which can result in inconsistencies and errors in the dataset. Among the studies analyzed, manual segmentation was indicated in 59 (92%). Even though a huge portion was performed with open datasets accomplished with structure masks, all of them were annotated manually by experts, leading to the same biases. 

The biases during network training were also significantly dependent on the reference used. Although prostate segmentation can be sufficiently performed without histological confirmation, reliable PCa contouring and stratification should be performed with reference, minimizing subjectivity. According to Alqahtani et al., biopsy might not be efficient in detecting more aggressive cancer cells or providing a representative sample of the entire prostate cancer [82]. This can result in ISUP grade and stage upgrade after radical prostatectomy. The study found that 31.6% of patients had an upgraded ISUP grade from a 12-core biopsy to a specimen on laparoscopic radical prostatectomy (LRP). Although fusion-guided biopsy can reduce this percentage [83], it is still challenging to accurately estimate the distribution and shape of prostate cancer. Among the 28 studies dedicated to PCa detection, stratification, and reconstruction, biopsy was used as a reference in 20 (71%). Biopsy is not relevant in PCa contouring because biopsy samples represent only a small area of the prostate gland and may not accurately reflect the location and extent of cancerous tissue within the gland [27]. 

Finally, validation and testing are important steps in developing and evaluating DL-based models for PCa diagnosis to ensure the accuracy and generalizability of their predictions. Validation helps identify any potential issues with overfitting, where the model has learned to perform well on the training data but does not generalize well to new data. Testing, on the other hand, is the process of evaluating a model’s performance on a completely new dataset that has not been previously seen by the model, providing its true generalizability. Among the papers analyzed in this review, all were accomplished using the results of internal validation. Testing was performed in 35 studies (55%), whereas external validation was performed in 10 papers. Interestingly, all studies investigating DL use for PET/CT Pca diagnosis provided testing.

However, the shortcomings of the proposed review are noteworthy. First, it was not systematic and we did not assess the quality of the selected studies. Instead, our goal was to analyze the current state of DL-based models by considering the challenges mentioned previously. Second, the search was limited to studies describing the use of DL in prostate reconstruction, the detection/stratification/reconstruction of prostate cancer, PET/CT PCa diagnosis, ADT therapy, and prostate biopsy, whereas there are plenty of papers dedicated to the use of DL in other aspects of prostate cancer diagnosis. For example, we focused on DL use in the context of prostate biopsy performance, not for histopathological investigation, as the latter is a separate topic dedicated to the goal of the review alone. Third, we did not analyze ML as a whole but only its part relevant to DL. 

## 5. Conclusions

DL models detecting prostate cancer on MRI show promise but are not yet ready for clinical use owing to variability in methods, labels, and evaluation criteria. These have mostly been developed on small datasets with high heterogeneity. Conducting additional research while acknowledging all the limitations outlined is crucial for reinforcing the effectiveness of DL-based models in clinical settings.

## Figures and Tables

**Table 1:** Summary of studies dedicated to the use of deep learning algorithms for three-dimensional prostate segmentation on MRI. Local and open datasets were used in 13 and 14 studies. Among the latter, Promise12, ProstateX, ISBI13, ASPS13, 12CVB, BWH, QIN-Prostate, and Decathlon datasets were used in 11, 3, 2, 1, 1, 1, 1, and 1 study, respectively. Datasets with magnetic field power of 3 T, 1.5 T, and 3/1.5 T were used in 18, 0, and 3 studies, respectively. Multi-vendor datasets were used in 16 studies. Median number of cases was 146 with minimum and maximum equal to 25 and 648. Among sequences, T2-weighted images (T2WIs), diffusion-weighted images (DWIs) and apparent diffusion coefficient (ADC) maps were used in 21, 1, and 2 studies, respectively. The segmentation of area of interest was performed manually in all cases (although most papers used open datasets with already annotated prostates, annotations were created manually by experts). All studies provided internal validation. The test was performed in 8 studies, whereas an external one was performed in 3 studies.

Authors	Network	Power of Magnetic Field, Tesla	Number of Institutional Datasets	Open Datasets Used	Vendors	Sequences	Number of Cases	Prostate Segmentation	Validity	Test	DSC
da Silva et al. [6]	coarse-to-fine segmentation DCNN	3	-	PROMISE12	Multi	T2WI	56	Manual	Internal	Internal	0.85
Wang et al. [7]	3D DSD-FCN	3	1	PROMISE12	Multi	T2WI	90	Manual	Internal	-	0.855
Liu et al. [8]	DDSP ConNet	3	-	PROMISE12	Multi	T2WI	80	Manual	Internal	-	0.8578
Nai et al. [9]	HighRes3DNet	3	-	ProstateX	Multi	T2WI, DWI, ADC	160	Manual	Internal	-	0.890
Yu et al. [10]	ConvNet	3	-	PROMISE12	Multi	T2WI	80	Manual	Internal	-	0.8693
Karimi et al. [11]	CNN with statistical shape model	3	1	PROMISE12	Multi	T2WI	75	Manual	Internal	-	0.88
Ushinsky et al. [12]	Hybrid 3D/2D U-Net	3	1	-	Single	T2WI	299	Manual	Internal	-	0.898
Yan et al. [13]	P-DNN	3	-	PROMISE12	Multi	T2WI	80	Manual	Internal	-	0.899
Jia et al. [14]	3D APA-Net	3/1.5	-	PROMISE12, ASPS13	Multi	T2WI	140	Manual	Internal	-	0.901
Comelli et al. [15]	E-Net	3	1	-	Single	T2WI	85	Manual	Internal	-	0.9089
Bardis et al. [16]	Hybrid 3D/2D U-Net	3	1	-	Multi	T2WI	242	Manual	Internal	Internal	0.940
Sanford et al. [17]	2D-3D hybrid CNN	3/1.5	5	-	Multi	T2WI	648	Manual	Internal	External	0.915
Liu et al. [18]	MS-Net	3/1.5	-	ISBI13, I2CVB	Multi	T2WI	79	Manual	Internal	Internal	0.9166
Wang et al. [19]	SegDGAN	3 T	1	Decathlon, ISBI13, QIN-PROSTATE, PROMISE12	Multi	T2WI	335	Manual	Internal	External	0.9166
Aldoj et al. [20]	Dense U-net	3	1	-	Single	T2WI	188	Manual	Internal	-	0.921
To et al. [21]	3D DM-net-8feat	3	1	PROMISE12	Multi	T2WI, ADC	280	Manual	Internal	Internal	0.9511
Zhu et al. [22]	BOWDA-Net	3	1	PROMISE12, BWH	Multi	T2WI	146	Manual	Internal	External	0.9254
Zhu et al. [23]	double 2D U-Net	3	1	-	Single	T2WI	163	Manual	Internal	-	0.927
Meyer et al. [24]	anisotropic 3D multi-stream CNN	3	1	ProstateX	Multi	T2WI	156	Manual	Internal	Internal	0.933
Chen et al. [25]	AlexNet	3	1	-	Single	T2WI	25	Manual	Internal	-	0.9768
Yan et al. [26]	PSPNet	3	-	PROMISE12	Multi	T2WI	270	Manual	Internal	-	0.9865

DCNN: deep convolutional neural network; 3D: three-dimensional; 2D: two-dimensional; DSD-FCN: deeply supervised densely fully convolutional network; DDSP: Differentiable Digital Signal Processing; CNN: convolutional neural network; P-DNN: Propagation Deep Neural Network, APA-Net: Adversarial Pyramid Anisotropic Convolutional Network; BOWDA-Net: boundary-weighted adaptive neural network; PSPNet: Pyramid Scene Parsing Network; T2WI: T2-weighted images; ADC: apparent diffusion coefficient; DWI: diffusion-weighted images; DSC: Dice similarity coefficient.

**Table 2:** Summary of studies dedicated to the use of deep learning in PCa detection and stratification on MRI. Local and open datasets were used in 16 and 11 studies. Among the latter, ProstateX is used in all studies, whereas Prostate-MRI, Prostate-Diagnosis, and TCGA-PRAD datasets were used in one study. Datasets with magnetic field power of 3 T, 1.5 T, and 3/1.5 T were used in 19, 1, and 2 studies, respectively. Multi-vendor datasets were used in 18 studies. The median number of cases was 344 with a minimum–maximum range of 37–2170 cases. Among sequences, T2WI, DWI, ADC, and DCE were used in 17, 13, 19, and 5 studies, respectively. In the datasets used, PCa was localized in PZ, TZ, AS, and SV in 13, 11, 8, and 8 studies, respectively. Notably, AS and SV localizations were provided only with the use of the ProstateX open dataset. As a reference, biopsy and whole-mount histopathology were used in 16 and 6 studies, respectively. Segmentation of area of interest was performed manually or semi-autonomously in 20 and 2 studies. In all studies, validation was performed with an internal dataset. Testing was carried out in 13 papers, whereas external data were used in 4 studies.

Authors	Network	Power of Magnetic Field, Tesla	Number of Institutional Datasets	Open Datasets Used	Different Scanners Vendors	Number of Cases	Segmentation Type	PCa Location	Reference	Sequences	Validity	Test	AUC
Ishioka et al. [27]	U-net + ResNet50	1.5	1	-	Multi	335	Manual	-	Biopsy	T2WI	Internal	-	0.645
Zabihollahy et al. [28]	Ensemble U-Net-based model	3	1	-	Single	226	Manual	PZ	Whole-mount histopathology	ADC	Internal	-	0.779
Mehrtash et al. [29]	9-layer 3D CNN	3	-	ProstateX	Multi	344	Manual	PZ, TZ, AFMS, SV	Biopsy	T2WI, DWI, ADC, DCE	Internal	Internal	0.80
Saha et al. [30]	Two parallel 3D CNNs	3	2	-	Multi	2137	Manual	PZ, TZ	Biopsy	T2WI, DWI, ADC	Internal	External	0.885
Chen et al. [31]	VGG-16	3	-	ProstateX	Multi	344	Manual	PZ, TZ, AS, SV	Biopsy	T2WI, DWI, DCE	Internal	Internal	0.83
Sobecki et al. [32]	3D VGG-16	3	-	ProstateX	Multi	344	Manual	PZ, TZ, AS, SV	Biopsy	T2, DWI, ADC, DCE	Internal	Internal	0.84
Sanyal et al. [33]	U-Net	3	1	-	Multi	77	Manual	PZ	Biopsy	DWI, ADC	Internal	-	0.86
Bhattacharya et al. [34]	CorrSigNet	3	1	-	Multi	95	Manual	-	Whole-mount histopathology	T2WI, ADC	Internal	Internal	0.86
Yu et al. [35]	Res-U-Net	3	7	ProstateX	Multi	2170	Semi-automated	-	Biopsy	T2WI, DWI, ADC	Internal	External	0.867
Yoo et al. [36]	ResNet	3	1	-	Single	427	Semi-automated	-	Biopsy	ADC	Internal	Internal	0.87
Zhong et al. [37]	ResNet	3	1	-	Multi	140	Manual	PZ, TZ	Whole-mount histopathology	T2WI, ADC	Internal	Internal	0.876
Khosravi et al. [38]	GoogLeNet	3/1.5	1	ProstateX, Prostate-MRI, Prostate-Diagnosis, TCGA-PRAD	Multi	400	Manual	-	Whole-mount histopathology	T2WI	Internal	Internal	0.89
Arif et al. [39]	12-layer CNN	3	1	-	Single	292	Manual	PZ, TZ	Biopsy	T2WI, DWI, ADC	Internal	-	0.89
Wang et al. [40]	TDN and dual-path CNN	3	1	ProstateX	Multi	360	Manual	-	Biopsy	T2WI, ADC	Internal	-	0.8978
Abdelmaksoud et al. [41]	VGGnet	3/1.5	1	-	Multi	37	Manual	-	Biopsy	ADC	Internal	-	0.91
Aldoj et al. [42]	12-layer 3D CNN	3	-	ProstateX	Multi	200	Manual	PZ, TZ, AS, SV	Biopsy	DWI, ADC, DCE	Internal	-	0.91
Song et al. [43]	Modificated VGGNet	3	-	ProstateX	Multi	195	Manual	PZ, TZ, AS, SV	Biopsy	T2WI, DWI, ADC	Internal	Internal	0.944
Pellicer-Valero et al. [44]	3D Retina U-Net	3	1	ProstateX	Multi	490	Manual	PZ, TZ, AS, SV	Biopsy	T2WI, DWI, ADC, DCE	Internal	Internal	0.95
Xu et al. [45]	ResNet	3	-	ProstateX	Multi	346	Manual	PZ, TZ, AS, SV	Biopsy	T2WI, DWI, ADC	Internal	-	0.97
Cao et al. [46]	FocalNet	3	1	-	Multi	417	Manual	-	Whole-mount histopathology	T2WI, ADC	Internal	-	0.81
Hou et al. [47]	PAGNet	3	2	-	Single	840	Manual	-	Whole-mount histopathology	T2WI, DWI, ADC	Internal	External	0.728
Zong et al. [48]	“Vanilla” VGG	3	1	ProstateX	Multi	367	Manual	PZ, TZ, AS, SV	Biopsy	T2WI, DWI, ADC	Internal	External	0.84

3D, three-dimensional; CNN, convoluted neural network; TDN, tissue deformation network; VGG, visual geometric group; PZ, peripheral zone; TZ, transition zone; AFMS, anterior fibromuscular stroma; SV, seminal vesicles; T2WI, T2-weighted images; ADC, apparent diffusion coefficient; DWI, diffusion-weighted imaging; DCE, dynamic contrast enhancement; AUC, area under the characteristic curve.

**Table 3:** Summary of studies dedicated to the use of deep learning for PCa 3D segmentation on MRI. Local and open datasets were used in 3 and 3 studies. Among the latter, ProstateX and 12 CVB were used in two and one study, respectively. Datasets with magnetic field power of 3 T, 1.5 T, and 3/1.5 T were used in 5, 0, and 1 study, respectively. Multi-vendor datasets were used in four studies. The median number of cases was 129, with a minimum–maximum range of 16–204 cases. Among sequences, T2WI, DWI, and ADC were used in 6, 4, and 5 studies, respectively. In the datasets used, PCa was localized in PZ, TZ, CZ, AS, and SV in 5, 2, 1, 2, and 2 studies, respectively. Again, AS and SV localizations were provided only with the use of the ProstateX open dataset. As a reference, biopsy and whole-mount histopathology were used in 4 and 2 studies, respectively. Segmentation of area of interest was performed manually or semi-autonomously in five and one study. In all studies, validation was performed with an internal dataset. Testing was carried out in 3 papers and only with internal data.

Authors	Network	Power of Magnetic Field, Tesla	Number of Institutional Datasets	Open Datasets Use	Different Scanners Vendors	Sequences	Segmentation Type	PCa Location	Reference	Number of Cases	Validity	Test	DSC
Gunashekar et al. [49]	3D U-Net	3/1.5	1	-	Multi	T2, DWI, ADC	Manual	-	Whole-mount histopathology	122	Internal	-	0.32
de Vente et al. [50]	2D U-Net	3	-	ProstateX	Multi	T2, ADC	Semi-automated	PZ, TZ, AS, SV	Biopsy	172	Internal	Internal	0.370
Lai et al. [51]	SegNet	3	-	ProstateX	Multi	T2, DWI, ADC	Manual	PZ, TZ, AS, SV	Biopsy	204	Internal	Internal	0.5273
Lee et al. [52]	SUconvGRU	3	1	-	Single	T2, DWI, DCE	Manual	PZ, TZ	Whole-mount histopathology	16	Internal	-	0.5323
Chen et al. [53]	2D U-Net	3	1	-	Multi	T2, DWI, ADC	Manual	PZ, TZ	Biopsy	136	Internal	Internal	0.6333
Alkadi et al. [54]	DCNN with modified VGG16	3	-	12CVB	Single	T2	Manual	PZ, TZ, CZ	Biopsy	19	Internal	-	0.892

3D, three-dimensional; 2D, two-dimensional; DCNN, deep convolutional neural network; VGG, visual geometric group; PZ, peripheral zone; TZ, transition zone; CZ, central zone; SV, seminal vesicles; T2WI, T2-weighted images; ADC, apparent diffusion coefficient; DWI, diffusion-weighted imaging; DCE, dynamic contrast enhancement; DSC, Dice similarity coefficient.

**Table 4:** Summary of studies dedicated to the use of deep learning for PET/CT diagnosis of PCa. All studies were performed with institutional datasets. Six of seven studies used data from a single institution. Among radiotracers, [^68^Ga]Ga-PSMA-11, [^18^F]DCFPyl, and [^18^F]PSMA-1007 were used in 5, 1, and 1 study, respectively. The median number of cases was 193 with a minimum–maximum range of 39–660 cases. During training, images were labeled semi-automatically or manually in 2 and 5 studies, respectively. Validity and tests are provided in all studies, whereas an external test was performed in one paper.

Authors	Network	Radiotracer	Number of Institutional Datasets	Open Datasets Use	Segmentation Type	Number of Cases	Validity	Test
Hartenstein et al. [55]	CNN	[^68^Ga]Ga-PSMA-11	1	-	Semi-automated	549	Internal	Internal
Capobianco et al. [56]	CNN	[^68^Ga]Ga-PSMA-11	1	-	Semi-automated	173	Internal	Internal
Ghezzo et al. [57]	CNN	[^68^Ga]Ga-PSMA-11	1	-	Manual	39	Internal	Internal
Kendrick et al. [58]	3D U-Net	[^68^Ga]Ga-PSMA-11	1	-	Manual	193	Internal	Internal
Leung et al. [59]	CNN	[^18^F]DCFPyl	1	-	Manual	267	Internal	Internal
Trägårdh et al. [60]	3D U-Net	[^18^F]PSMA-1007	1	-	Manual	660	Internal	Internal
Zhao et al. [61]	2.5D U-Net	[^68^Ga]Ga-PSMA-11	3	-	Manual	193	Internal	External

3D, three-dimensional; CNN, convoluted neural network; Ga, gallium; PSMA, prostate-specific membrane antigen; F, fluorine; [^18^F]DCFPyl: Piflufolastat F-18.

**Table 5:** Summary of studies dedicated to the use of deep learning in the context of ADT for the PCa. Only two studies fit inclusion criteria. Both are performed with single-institution dataset only and with the manual labeling of training data. Validity is provided by two papers, whereas an internal test is only provided in one study. As an input, DP is used in both; however, one study used clinical signs and radiology bone scans in addition. Among studies is a tremendous gap in the number of cases included: 5727 versus 154.

Authors	Network	Input	Number of Institutional Datasets	Open Datasets Use	Segmentation Type	Number of Cases	Validity	Test
Spratt et al. [62]	Res-Net	DP	1	-	Manual	5727	Internal	-
Mobadersany et al. [63]	SCNNs1 and CPH	Clinics + DP + rBS	1	-	Manual	154	Internal	Internal

SCNNs1, survival convolutional neural networks; CPH, Cox proportional-hazards model; Res-Net: Residual Neural Network; DP, digital pathology; rBS, radiology bone scan.

**Table 6:** Summary of studies dedicated to the use of deep learning in the context of prostate biopsy. Three and three studies were conducted with a single- and multi-institutional dataset, respectively. Among the latter, the number of institutes from which data were included was 2, 3, and 29. TeUS, TRUS, and MRI were used as input modalities in 2, 3, and 1 study, respectively. All studies used manual labeling when training networks. Four papers used biopsy as a groundtruth. The median number of cases was 172 with a minimum–maximum range of 124–905 cases. Validity and testing were performed in 6 and 3 studies, respectively.

Authors	Network	Modality	Number of Institutional Datasets	Open Datasets Use	Segmentation Type	Groundtruth	Number of Cases	Validity	Test
Sedghi et al. [64]	DNM	TeUS	1	-	Manual	Biopsy	157	Internal	-
Azizi et al. [65]	Res-Net	TeUS	2	-	Manual	Biopsy	163	Internal	External
Van Sloun et al. [66]	U-net	TRUS	3	-	Manual	-	181	Internal	-
Orlando et al. [67]	2D U-net	TRUS	1	-	Manual	Biopsy	206	Internal	Internal
To et al. [68]	DNN	TRUS	1	-	Manual	-	124	Internal	-
Soerensen et al. [69]	ProGNet	MRI	29	-	Manual	Biopsy	905	Internal	External

DNM, deep neural mapping; Res-Net: Residual Neural Network; 2D, two-dimensional; DNN, deep neural network; TeUS, temporal ultrasound; TRUS, transrectal ultrasound; MRI, magnetic resonance imaging.
